# MAPping the Ndc80 loop in cancer: A possible link between Ndc80/Hec1 overproduction and cancer formation

**DOI:** 10.1002/bies.201400175

**Published:** 2015-01-02

**Authors:** Ngang Heok Tang, Takashi Toda

**Affiliations:** Laboratory of Cell Regulation, Cancer Research UK, London Research Institute, Lincoln's Inn Fields LaboratoriesLondon, UK

**Keywords:** cancer, ch-TOG, Kinesin-8, loop, Ndc80/Hec1, overexpression, TACC

## Abstract

Mis-regulation (e.g. overproduction) of the human Ndc80/Hec1 outer kinetochore protein has been associated with aneuploidy and tumourigenesis, but the genetic basis and underlying mechanisms of this phenomenon remain poorly understood. Recent studies have identified the ubiquitous Ndc80 internal loop as a protein-protein interaction platform. Binding partners include the Ska complex, the replication licensing factor Cdt1, the Dam1 complex, TACC-TOG microtubule-associated proteins (MAPs) and kinesin motors. We review the field and propose that the overproduction of Ndc80 may unfavourably absorb these interactors through the internal loop domain and lead to a change in the equilibrium of MAPs and motors in the cells. This sequestration will disrupt microtubule dynamics and the proper segregation of chromosomes in mitosis, leading to aneuploid formation. Further investigation of Ndc80 internal loop-MAPs interactions will bring new insights into their roles in kinetochore-microtubule attachment and tumourigenesis.

## Introduction

**Figure 1:**
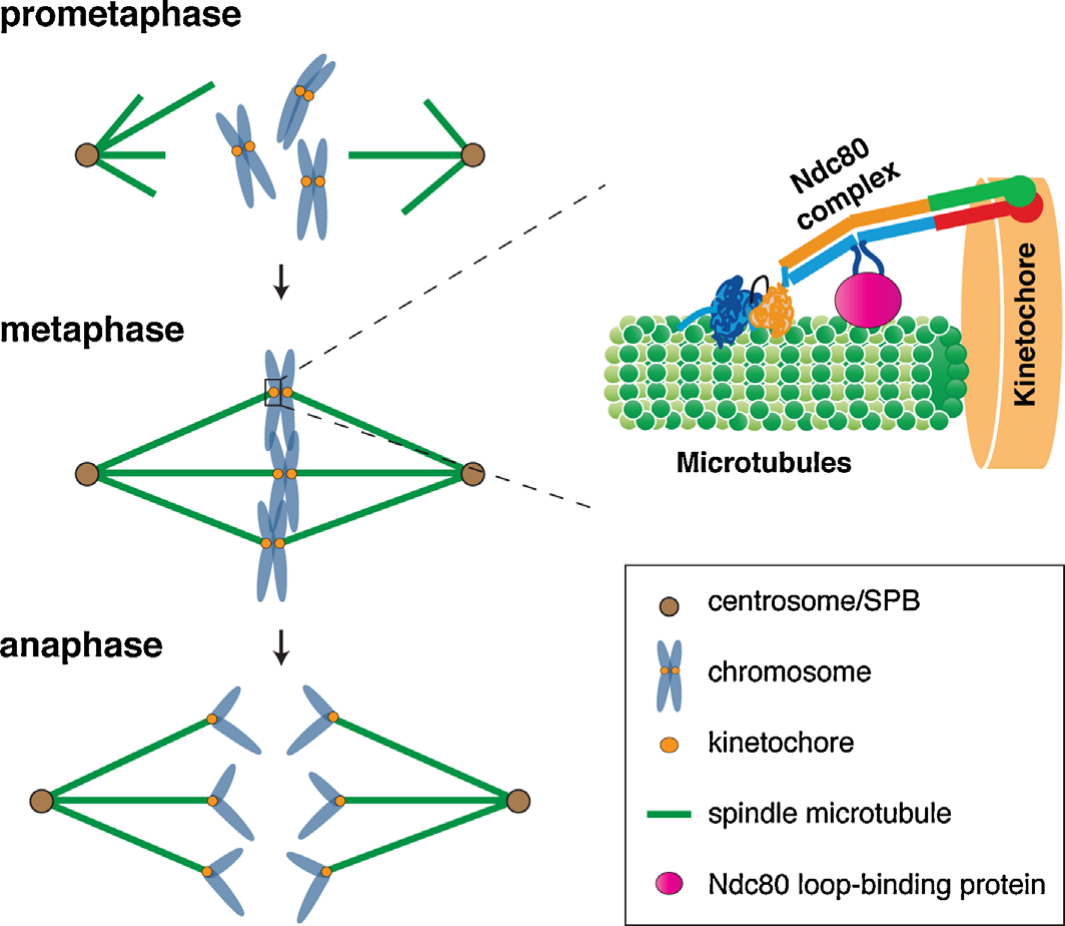
Chromosome segregation during mitosis. Left: The diagram summarises chromosome movements in different stages of mitosis. In prometaphase, spindle microtubules emanate from the centrosome/SPB to bind to chromosomes at the kinetochore region. Attached chromosomes are aligned in the metaphase plate during metaphase. Sister chromatids are segregated apart towards the opposite poles during anaphase. Right: Ndc80 attaches to the spindle microtubule through its N-terminal tail. In addition, the internal loop region of Ndc80 binds to different proteins to regulate proper spindle-microtubule attachment.

Mitosis plays a central part in accurate segregation of genetic materials (i.e. chromosomes) into the two daughter cells (Fig. 1). Chromosome missegregation results in genome instability such as aneuploidy, which is a hallmark of cancer. Mis-regulation in the form of up- or down-regulation of the genes/proteins involved in microtubule (MT) dynamics, mitotic checkpoint or kinetochore-MT attachment are known to cause chromosome instability (CIN) [1,2]. Examples include the colonic and hepatic tumour overexpressed gene (ch-TOG) [3] and the genes encoding the transforming acidic coiled-coil proteins (TACCs) [4], spindle assembly checkpoint (SAC) proteins Mad2 [5] and Bub1 [6], the Ska kinetochore complex [7], kinesin-8 motors [8,9], and the outer kinetochore protein Ndc80/Hec1 (Highly Expressed in Cancer) [10–12], which are all upregulated in several cancers. Although overexpression of these genes may not have direct causative effects on the proliferative nature of cancerous cells, these studies suggest a correlation between increased expression of mitotic genes and cancer formation. Whilst these results are consistent with the idea that overproduction of these proteins is linked to tumourigenesis, there is also a view opposing this, supported by examples such as the absent or reduced expression of TACCs in ovarian and thyroid cancer tissues [13]. In addition, Mad2 and BubR1 confer tumour-suppressor activities [14,15]. Overall, previous and current studies strongly suggest a functional relationship between the stoichiometry of kinetochore proteins, SAC components and MT-associated proteins (MAPs) and tumourigenesis.

## Hypothesis

Despite several previous studies on the collaborative relationships between mitotic gene expression and cancer, the mechanistic details of tumourigenesis induced by coordinated transcriptional up-regulation remain poorly understood. Here, we propose a new hypothesis for this phenomenon: elevated levels of a mitotic protein will sequester away (‘absorb’) its binding partners, thereby disrupting the protein equilibrium during mitosis and consequently leading to abnormal mitotic progression and chromosome missegregation. To compensate for the loss of this protein equilibrium, expression of genes encoding the kinetochore, MAPs and SAC components may subsequently be altered (i.e. up- or down-regulated). This scenario at least in part accounts for how mis-regulation of a cluster of mitotic genes is observed in various cancer cells.

## Many mitotic proteins are genetically and functionally related to each other

A myriad of structural and regulatory factors are involved in mitosis to ensure faithful chromosome segregation into two daughter cells. Mitotic proteins often genetically, functionally and physically interact with each other. Here, we provide two examples – Ndc80 and the TACC proteins, and show how mis-regulation (mutation or deletion) of these proteins results in mitotic defects leading to aneuploid formation. We will focus on the recent advances made in the fission yeast *Schizosaccharomyces pombe* and human cell lines that support our proposed hypothesis (see Table1 for summary of protein nomenclatures and functions).

**table 1:** List of proteins and their functions discussed in this review/hypothesis

Human	Fission yeast	Budding yeast	Functions
Ndc80/Hec1	Ndc80	Ndc80	A component of the Ndc80 outer kinetochore complex
ch-TOG	Alp14 Dis1	Stu2	Microtubule (MT) polymerase
TACC1, 2	—	—	MT-associated proteins (MAPs), localise to the centrosome
TACC3	Alp7	Slk19*	MAP, localises to the centrosome/SPB, spindle MT and the kinetochore
Cdt1	Cdt1	Cdt1/TAH11	DNA replication licensing factor
Ska complex (Ska1,2,3)	—	—	Binds to the MT and localises to the kinetochore
—	Dam1	Dam1	A component of the Dam1 complex that binds to the MT and localises to the kinetochore (Assumed to be the yeast homolog of the Ska complex)
Kif18A	Klp5 Klp6	Kip3	Kinesin 8, regulates MT dynamics (Kip3 is a MT depolymerase)

* Slk19 is a kinetochore protein, but unlike humans and fission yeasts, its binding to Stu2 has not been reported [100,101].

### The Ndc80/Hec1 outer kinetochore protein

**Figure 2:**
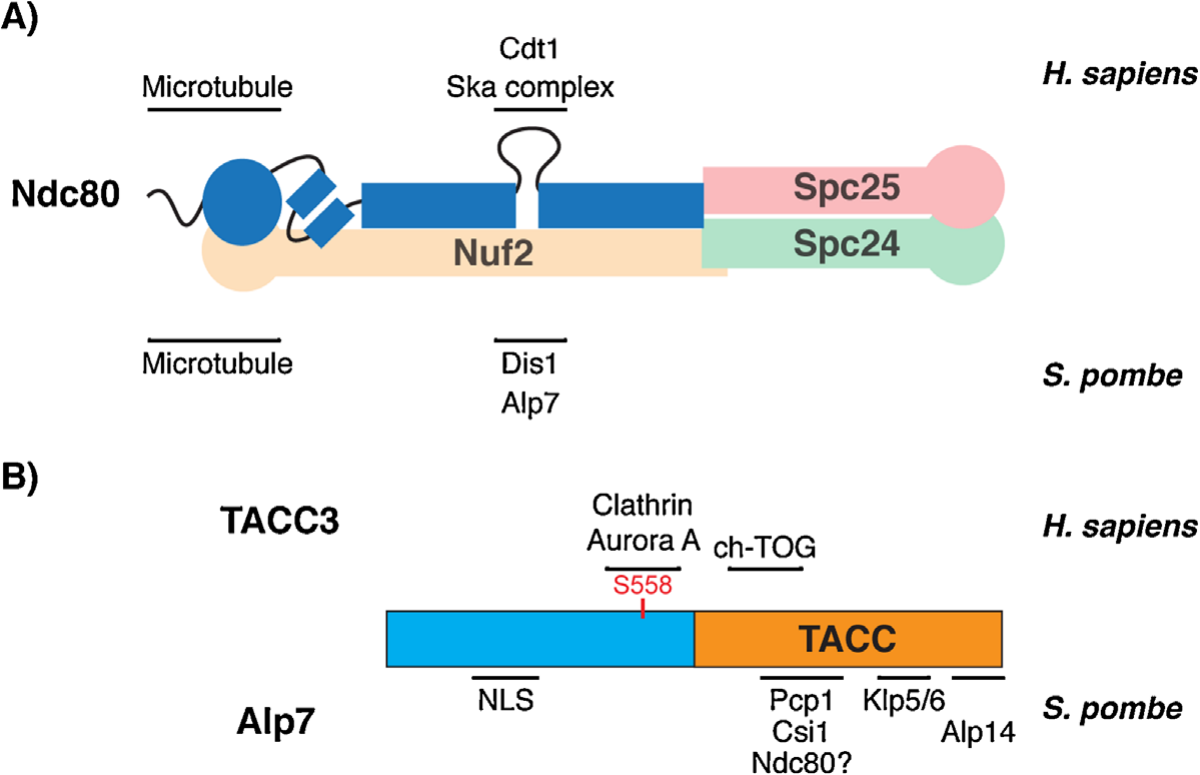
Binding partners of Ndc80 and TACC proteins. The diagram summarises binding partners of (A) Ndc80 and (B) TACC proteins in *H. sapiens* and *S. pombe*. A: Ndc80 forms a heterotetramer complex with Nuf2, Spc24 and Spc25. The N-terminal tail and Calponin-homology (CH) domain of Ndc80 bind directly to microtubules. In *H. sapiens*, the Ndc80 internal loop binds to Cdt1 and Ska1, whereas the loop binds to Dis1 and Alp7 in *S. pombe*. Note that the component of the Ska complex that interacts with the loop remains to be established. B: In *H. sapiens*, S558 (depicted in red) of TACC3 is phosphorylated by Aurora A. This phosphorylation is important for clathrin binding activity of TACC3. The TACC domain of TACC3 binds to ch-TOG. In *S. pombe*, the N-terminal of Alp7/TACC contains a nuclear localisation signal (NLS). The TACC domain of Alp7 binds to Pcp1 and Csi1 for SPB-targeting and Alp14 for microtubule localisation. The TACC domain is also deemed to bind to Ndc80, using the similar region responsible for Pcp1 and Csi1 binding.

Ndc80 binds to Nuf2, Spc24 and Spc25, forming a dumbbell-shaped complex of ∼57 nm in length with two globular heads connected through a long coiled-coil region (Fig. 2A) [16–19]. This complex localises to the outer kinetochore and directly binds to MTs through the N-terminal tail and calponin-homology (CH) domain of Ndc80 [20–23] (Fig. 1). A number of biochemical studies have identified several phosphorylation sites within this N-terminal region of Ndc80. Aurora B kinase, which localises to the inner centromere region, has been shown to phosphorylate Ndc80, both in vitro [18,24,25] and in vivo [25,26]. This phosphorylation results in deterioration of the Ndc80-MT interaction, thereby interfering with the spatiotemporal control of kinetochore-MT attachment. Non-stabilised kinetochore-MT attachment (e.g. incorrect attachment) leads to SAC activation and mitotic delay. Once mal-attachment is corrected, tension is exerted to the kinetochore and anaphase initiates, leading to chromosome segregation [27–29]. It is worth noting that a recent study using the budding yeast *Saccharomyces cerevisiae*, on the other hand, has challenged this model for which Aurora B localisation to the inner centromere is a prerequisite [30]. An alternative model proposes that tension sensing and consequent chromosome biorientation are successfully established via Aurora B that localises to the mitotic spindle, not the inner centromere, as long as Aurora B is activated [30]. In addition to Aurora B, phosphorylation of the budding yeast Ndc80 by Mps1 has been implicated in SAC signalling [31] and human Ndc80 phosphorylation (at serine 165 in the CH domain) by Nek2 kinase plays a critical role in faithful chromosome segregation [32].

Intriguingly, ultrastructural studies of the reconstituted budding yeast Ndc80 complex have revealed the presence of a kink (or loop) in the middle of the internal coiled-coil region within the Ndc80 protein [33–35]. More recently, the kink/loop has also been identified in human Ndc80 [36,37]. This loop was originally proposed to provide flexibility to the complex, allowing an angular bending of the two flanking rods, by which it potentially serves as a tension-sensing mechanism at the kinetochore. Although this notion remains promising [38–40], several recent studies in different organisms have identified the unexpected roles of the Ndc80 loop as a protein-protein interaction motif. To date, the Ndc80 loop has been found to associate with the Dam1 MT-binding kinetochore complex in *S. cerevisiae* [41], Dis1/TOG and Alp7/TACC-Alp14/TOG MAPs in *S. pombe* [42,43], and the Cdt1 licensing factor and the Ska kinetochore complex in human cell lines [36,44]. Extensive reviews on the roles of the Ndc80 loop can be found in [45,46].

In *S. pombe*, localisation of Dis1 to the kinetochore through the Ndc80 loop stabilises the mitotic spindle at the kinetochores [42]. This binding in turn allows loading of the Alp7-Alp14 complex to the Ndc80 loop to ensure faultless chromosome segregation in mitosis [43]. Our recent study has shown that the binding between the Ndc80 loop and Alp7-Alp14 is crucial for subsequent recruitment of the kinesin 8-protein phosphatase I complex (Klp5-Klp6-PP1) to the kinetochore [47]. This recruitment plays critical roles in ensuring timely mitotic progression and anaphase chromosome movement. In human cells, the Ndc80 internal loop recruits the Cdt1 licensing protein to facilitate stable kinetochore-MT attachment [44]. Furthermore, recruitment of the Ska complex to the Ndc80 internal loop plays important roles in the establishment of end-on kinetochore-MT binding and promotion of mitotic exit [36,48]. Taken together, these studies imply that by binding to various mitotic factors (Fig. 2A), the Ndc80 internal loop provides an indirect MT-binding domain in the Ndc80 complex and plays multiple roles in chromosome segregation, including the establishment of bipolar kinetochore-MT attachment and proper mitotic progression.

### The TACC and TOG proteins

TACC family proteins, characterised by the presence of the TACC domain in their C-termini, are conserved from yeast to humans. Three isoforms (TACC1-3) encoded by different genes have been identified in humans [4,49], whilst only one has been found in other organisms (Alp7/Mia1 in *S. pombe*; TAC-1 in *C. elegans*, Maskin/TACC3 in *X. laevis* and D-TACC in *D. melanogaster*) [50–53]. Several mitotic proteins, including ch-TOG, Aurora A kinase and clathrin, have been identified as TACC interactors [52,54–61]. In *S. pombe*, the Alp7/TACC-Alp14/TOG complex localises to the spindle pole body (SPB, the functional equivalent of the animal centrosome) to regulate bipolar spindle assembly and dynamics during mitosis [52,62]. Recent studies have identified two SPB components, the pericentrin-like Pcp1 and Csi1, as recruiters of the Alp7-Alp14 complex to the SPB [63,64]. In addition, the Alp7-Alp14 complex also plays critical roles at the kinetochore through interaction with the Ndc80 internal loop (see above) [43]. In human cell lines, formation of the TACC3-ch-TOG complex plays important roles in spindle pole organisation and the regulation of centrosomal MTs [65]. In addition, binding of TACC3 to clathrin allows localisation of this complex onto the kinetochore MTs, thereby stabilising the kinetochore fibres by inter-MT bridging [54,56]. It is also reported that this complex is critical for centrosome integrity and bipolar spindle formation [59,61,66]. Collectively, these studies indicate that TACC proteins may well act as a hub to interact with multiple proteins to play distinct roles in different stages during mitosis (Fig. 2B).

The XMAP215/ch-TOG family proteins are conserved from yeast to humans, and are characterised by the presence of TOG domains in the N-terminal region. The number of TOG domains present varies amongst different organisms: two in Stu2 (*S. cerevisiae*), Dis1 and Alp14 (*S. pombe*), three in Zyg9 (*C. elegans*), and five in Msps (*D. melanogaster*), XMAP215 (*X. laevis*) and ch-TOG (*H. sapiens*) [67,68]. Several lines of evidence have shown that XMAP215/ch-TOG proteins possess MT polymerase activities [68–72]. Recent structural studies have solved the structure of TOG domains, giving further insights into how these domains recognise and bind to free α/β tubulin dimers [73–77]. These studies report that both TOG1 and TOG2 from Stu2 [73,74] or TOG1-4 from Msps [77] bind preferentially to curved α/β tubulin dimers, thereby selectively recognising the growing MT ends and facilitating polymerisation of the MTs. Intriguingly, *S. pombe* cells contain two XMAP215/ch-TOG members – Dis1 and Alp14 [78–81], which have both been shown to localise to the kinetochore through the Ndc80 internal loop domain (see above) [42,43]. Cumulatively, not only do the XMAP215/TOG family proteins regulate MT dynamics through the TOG domains, but they also play crucial roles in the regulation of kinetochore-MT attachment and mitotic progression.

## Does overproduction of Ndc80 in fission yeast recapitulate analogous defects seen in human cancerous cells?

**Figure 3:**
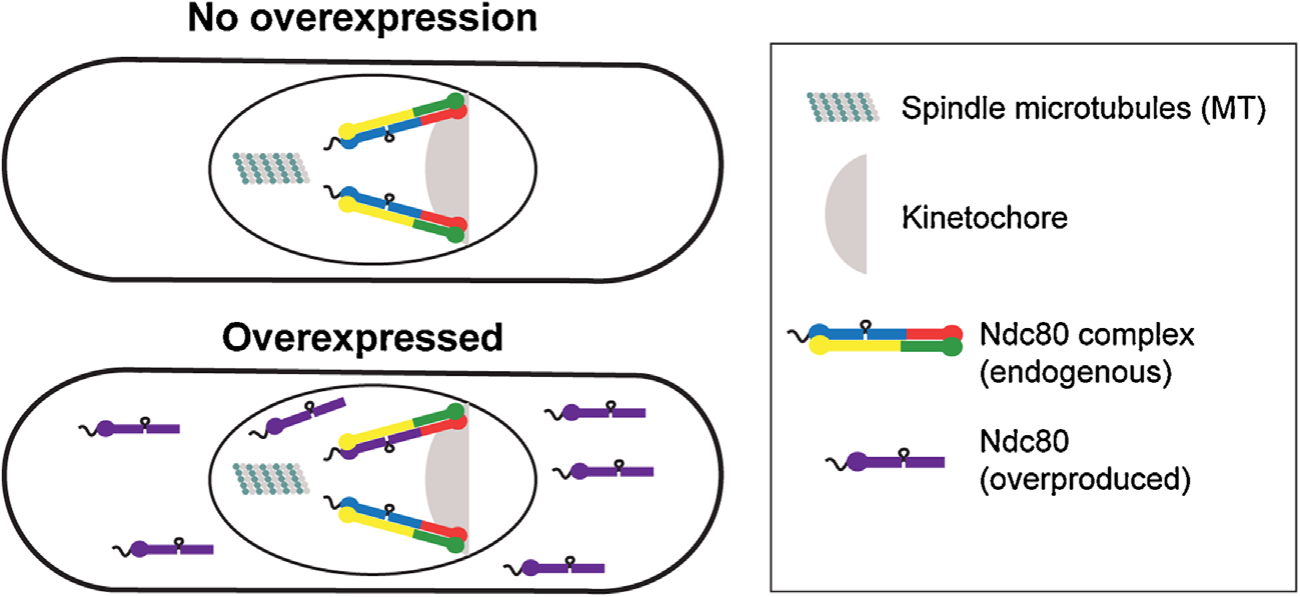
Overproduction of Ndc80 in *S. pombe* cells. The outer kinetochore Ndc80 complex is made up from Ndc80, Nuf2, Spc24 and Spc25. Under ‘overexpressed’ condition, two populations of Ndc80 will be formed. One propotion will be incorporated into the Ndc80 complex in place of endogenously produced Ndc80 and localise to the outer kinetochore, whereas the excess ‘free’ Ndc80 may be dispersed or form large polymers/aggregates in the cytoplasm.

Based on these studies, we propose that overproduced mitotic proteins will result in dominant negative sequestration of their interactors, and subsequently interfere with the normal functions of these proteins [82]. This spatial segregation may then elevate gene expression of the interactors, causing a coordinated up-regulation of a cluster of genes. We used Ndc80 as an example to test our hypothesis, by overproducing different Ndc80 constructs in *S. pombe* cells. Previous studies have shown that approximately 20–25 Ndc80 complexes bind to each kinetochore MT [83,84]. Fission yeast contains three chromosomes, in which each sister kinetochore attaches to two or three MTs [85,86], and the total copy number of the Ndc80 complex per cell is 500 to 1,600 molecules [87,88]. We envision that overproduced Ndc80 will generate an excess Ndc80 pool that cannot be incorporated into the complex, yet this ‘free’ Ndc80 population will nonetheless, bind to its binding partners (Fig. 3).

**Figure 4:**
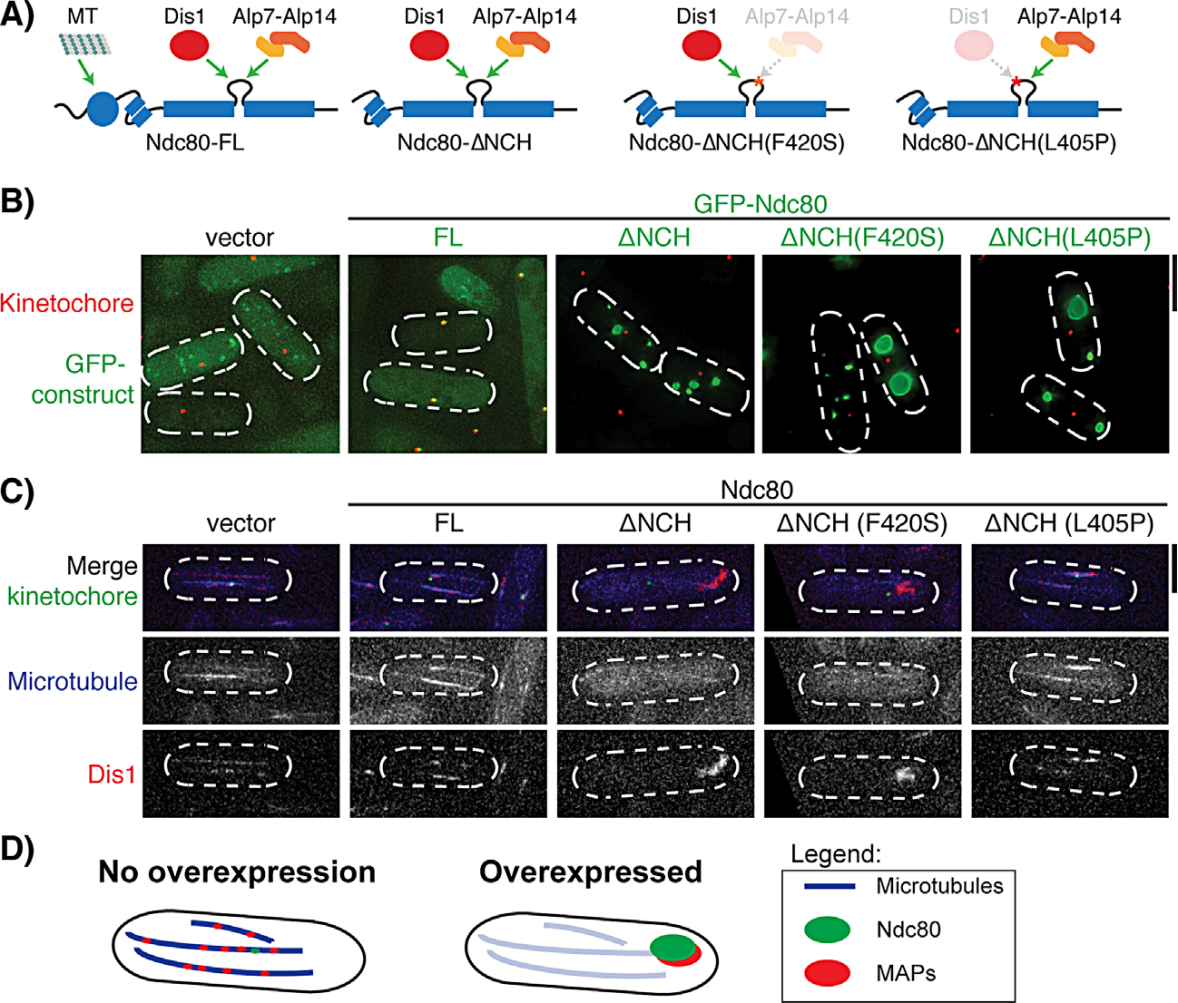
Excess ‘free’ Ndc80 absorbs Dis1 in the cytoplasm. A: A schematic showing the ability of different Ndc80 constructs to bind to microtubule (MT), Dis1 and Alp7-Alp14 complex. B: Localisation of overproduced GFP-Ndc80. Overproduced Ndc80-FL colocalise with the kinetochore (marked by Nuf2-mCherry). In sharp contrast, overproduced Ndc80-ΔNCH, Ndc80-ΔNCH(F420S) or Ndc80-ΔNCH(L405P) does not localise to the kinetochore, instead forming large polymers/aggregates in the cell. Scale bar, 5 µm. C: Localisation of Dis1 in cells overexpressing different Ndc80 constructs. Dis1 localises to the cytoplasmic MTs (marked by CFP-Atb2) during interphase (in vector, Ndc80-FL). Localisation of Dis1 to the cytoplasmic MTs is disrupted in cells overproducing Ndc80-ΔNCH or Ndc80-ΔNCH(F420S). This disruption is rescued by introducing L405P (defective in Dis1 binding) into the internal loop of Ndc80-ΔNCH. Scale bar, 5 µm. D: A schematic showing absorption of MAPs by overproduced Ndc80. Under normal conditions, Ndc80 localises to the kinetochore whereas MAPs localise to the MTs. When Ndc80 is overproduced, MAPs are sequestered by overproduced Ndc80, thereby disrupting the normal functions of MAPs (depicted by dimmer MTs).

As previously mentioned, the internal loop region of Ndc80 has been shown to be an important protein-protein interacting platform [45,46], so we retained the presence of the internal loop in each of our constructs. To this end, we created four different Ndc80 constructs, namely Ndc80-FL (full-length), Ndc80-ΔNCH, Ndc80-ΔNCH(F420S) and Ndc80-ΔNCH(L405P). Ndc80-FL binds to MTs through its N-terminal tail and CH domain (Fig. 4A) [20–23]. This MT-binding region is truncated in the Ndc80-ΔNCH constructs. We also introduced either the mutation F420S (defective in Alp7-binding) or L405P (defective in Dis1-binding) in the Ndc80-ΔNCH constructs to create Ndc80-ΔNCH(F420S) and Ndc80-ΔNCH(L405P), respectively (Fig. 4A). These constructs were then cloned into the pREP1 (or pREP41-GFP) vector under control of the inducible *nmt1* (or *nmt41*) promoter [89]. In the presence of thiamine, gene expression from the *nmt* promoter is repressed, whilst removal of thiamine from the medium allows high-level expression of the *ndc80* constructs.

## Overproduced Ndc80 sequesters its binding partner in the cells

We first examined the localisation patterns of the individual Ndc80 constructs. Ectopically produced Ndc80-FL colocalises with the kinetochore, whilst the Ndc80-ΔNCH constructs fail to localise to the kinetochore (Fig. 4B). This observation is consistent with a recent study showing the importance of the CH domain in the hetero-dimerisation of Ndc80 and Nuf2 [38]; without the function of this domain, Ndc80 cannot be incorporated into the Ndc80 complex. Intriguingly, we observed the formation of large polymers/aggregates in cells overproducing the Ndc80-ΔNCH constructs (Fig. 4B). This localisation pattern is highly unusual, suggesting a potential self-polymerisation activity of the constructs lacking the NCH domain. We did not however observe large polymers in cells overexpressing Ndc80-FL. We consider two possibilities to explain this observation. Firstly, Ndc80-ΔNCH fails to localise to the kinetochore, thus all the Ndc80-ΔNCH overproduced is freely available to bind to other MAPs and disrupt their functions. In contrast, Ndc80-FL forms a complex with endogenous Nuf2 and localises to the kinetochore. This localisation may have a dilution effect on the overproduced Ndc80-FL and reduce the population of excess ‘free’ Ndc80 present in the cells. Secondly, we envision that the absence of the NCH domain in Ndc80-ΔNCH may lead to non-physiological large polymers/aggregates in the cells by an unknown mechanism. These possibilities are not exclusive, and further experiments are required to test this notion.

Having observed the distinct localisation patterns of each individual Ndc80 construct, we then examined the localisation of Dis1, a known interacting partner of Ndc80 through the internal loop region [42]. As expected, Dis1 localises to the cytoplasmic MTs during interphase in cells expressing empty vectors or Ndc80-FL (Fig. 4C). Intriguingly, Dis1 forms large polymers/aggregates in cells expressing Ndc80-ΔNCH (Fig. 4C). Although colocalisation between Ndc80-ΔNCH and Dis1 is not formally proven, this localisation pattern is similar, if not identical, to that of Ndc80-ΔNCH. Further experiments on colocalisation or binding between Ndc80-ΔNCH and Dis1 should clarify this phenomenon. We noted that MT intensity is reduced in cells overexpressing Ndc80-ΔNCH, suggesting a disrupted MT structure possibly ascribable to malfunctioning of aggregated Dis1. We also observed similar localisation in cells expressing Ndc80-ΔNCH(F420S), consistent with the notion that the F420S mutation does not disrupt the ability to bind to Dis1 in the cells, but instead is defective in binding to the Alp7/TACC-Alp14/TOG complex [43]. In fact, localisation of Alp7 is not noticeably altered under the same condition, the reason for which is currently not explored further (unpublished). Interestingly, the defective phenotypes were rescued by introducing into the construct the L405P mutation with impaired Dis1 binding activity (Fig. 4C) [42]. In line with this observation, Dis1 localises on the MTs in the L405P mutant, in a similar manner to that seen in cells containing empty vectors or overproducing Ndc80-FL. Overall, these experiments strongly suggest that overproduced Ndc80 absorbs its binding partners (e.g. Dis1), thereby disrupting their functions (Fig. 4D). It would be of interest to test whether overproduction of other regions within Ndc80, including the N-terminal tail/CH domain and the C-terminal coiled coil domain could also sequestrate their individual interactors.

## Can in vivo overexpression be used as a tool to identify interacting partners?

We have shown that overproducing Ndc80-ΔNCH results in large aggregates in the cells, and that these aggregates abduct its interacting partner Dis1 (Fig. 4). This ability to form large aggregates suggests that the coiled-coil and loop domains of Ndc80 have the potential to self-polymerise, and that this polymerisation may play important roles in the functions of Ndc80. Furthermore, overexpression of the TACC domain in HeLa cells produces large polymers that appear to associate with MTs and tubulin in the cytoplasm [90]. It would be intriguing to test whether other TACC interactors such as ch-TOG and clathrin are also sequestered under these conditions. As the formation of large aggregates is easily observable and recognisable in the cells, we propose that this in vivo protein overproduction system could provide an excellent tool to identify the interacting partners of a protein (e.g. coimmunoprecipitation followed by mass spectrometry analysis). It is of note that overproduction of a protein may lead to unspecific binding of other functionally non-related proteins. Therefore, further in vivo validations are required upon the identification of interacting partners using this method.

The Ndc80 internal loop has been shown to bind several proteins in different organisms, but the Ndc80 loop-interacting partners known to date do not share homology amongst these organisms. For example, it is unknown whether TACC-TOG binds to the Ndc80 internal loop in vertebrates, though it is firmly established that TACC-TOG localisation to the Ndc80 internal loop in fission yeast plays important roles in mitosis [42,43] and meiosis [91]. We could now use this in vivo overproduction system to identify interacting partners of Ndc80 in different model organisms. We envision that this system could also be applied to identify interactors of other mitotic proteins.

## A new hypothesis: How does Ndc80 overproduction cause cancer?

Overexpression of Ndc80/Hec1 has long been implicated in tumourigenesis [10–12]. Interestingly, the expression of Ndc80/Hec1-associated genes (i.e. Nuf2, Spc24, Spc25 and Nek2) is coordinately up-regulated in Ndc80/Hec1-overexpressed cancer cells [92]. Recent analyses of gene expression profiles in cancer patients have suggested that altered expression of individual kinetochore genes is unlikely to cause cancer on its own [93]. Instead, it is proposed that these altered expression patterns in cancer cells arise as a consequence of an altered cell division programme. These studies indicate that the overexpression of a cluster of genes, rather than a single gene, affects the potential and severity of tumourigenesis. Nonetheless, it is possible that this coordinated expression of mitotic genes does promote the process of tumourigenesis, or contributes to the functional compensation for up-regulation of some mitotic proteins such as Ndc80. In line with this notion, overexpressing Ndc80 in mice displays increased levels of Mad2, leading to checkpoint hyperactivation and aneuploidy [12]. Furthermore, breast cancer patients with both Ndc80 and Nek2 overexpression display shorter survival compared to patients with only either Ndc80 or Nek2 overexpression [94]. In fission yeast, we observed prolonged mitotic arrest in cells overproducing Ndc80-ΔNCH or Ndc80-ΔNCH(F420S), suggesting that the SAC is continuously active in these cells. These data support the notion that overexpression of a single gene may induce similar overexpression of its genetic or functional partners, thereby leading to tumourigenesis.

**Figure 5:**
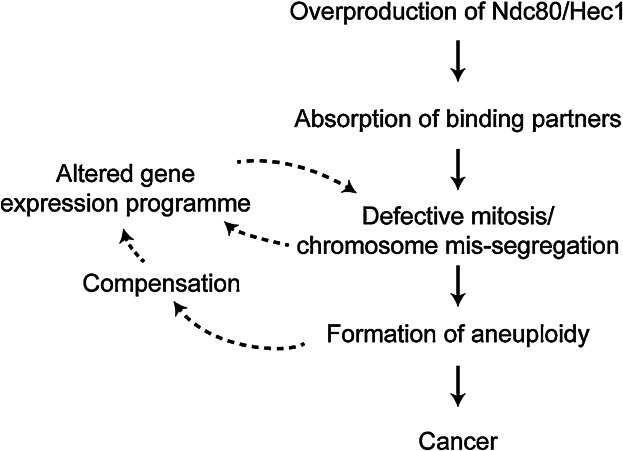
The proposed outcome of Ndc80/Hec1 overproduction. Overproducing Ndc80/Hec1 leads to the absorption of its interacting proteins by binding to the internal loop. This sequestration impedes mitotic progression and leads to chromosome mis-segregation, resulting in the formation of aneuploid progenies. Aneuploidy will, on one hand, promote tumourigenesis and yet on the other hand, trigger the cellular compensation, by which the transcription programme may alter in response to mitotic defects. Altered gene expression profiles, either up- or down-regulation of functionally related genes/proteins, will further promote or suppress growth of aneuploid cells. Cancer cells may arise from some of these populations.

In brief, we hypothesise that overproduced Ndc80/Hec1 may sequestrate its binding partners through the Ndc80 internal loop domain. This absorption will result in altered mitotic progression and defective chromosome segregation, leading to aneuploidy. On the other hand, in response to these defects, the transcription programme of Ndc80 and its interacting partners may also be altered as a result of different cell cycle profiles and/or cellular compensatory mechanism. This alteration may further promote growth of aneuploid cells (summarised in Fig. 5).

## Ndc80 and TACC proteins as potential drug targets

Due to their roles in controlling mitotic progression and chromosome segregation, both Ndc80/Hec1 and TACC proteins are considered potential targets for cancer drug development. The use of single vector-expressed short-hairpin RNAs (shRNAs) has shown that the depletion of Ndc80/Hec1 significantly reduces tumour size in mice [95]. Additionally, a small compound INH-1 that disrupts the Ndc80-Nek2 interaction has been developed [96] along with other INH-1 derivatives identified to show improved potency and efficacy [92,94,97]. Introducing such compounds to the cells leads to mitotic catastrophe and halts tumour progression. These studies indicate that the disruption of Ndc80 functions could provide promising therapeutic effects for cancer patients. On the other hand, a recent study has shown that depletion of TACC3 in lymphoma cells causes multi-polar spindle formation that subsequently leads to mitotic arrest and apoptosis [98]. The same group has then developed a small molecule, spindlactone (SPL) that inhibits the functions of the TACC-TOG complex [99]. Oral administration of SPL significantly reduces the tumour volume in mice [99], demonstrating the potential of SPL as a therapeutic agent for cancer chemotherapy in clinical applications.

## Clinical perspectives of the hypothesis

Recent advancements in drug development against the Ndc80/Hec1 and TACC proteins seem promising for clinical use in cancer treatment. However, several key features remain unexplored. Here we propose a few questions that could be addressed in the future to provide a better understanding of a functional relationship between gene overexpression and cancer formation. For simplicity, we focus on Ndc80/Hec1 and TACC; these questions could also be applied to other mitotic genes/proteins.

### Does overexpression of Ndc80 induce overexpression of its genetic and functional partners?

Recent studies have identified several proteins that interact with the Ndc80 internal loop [45,46], and we have shown that the overproduction of Ndc80 results in absorption of its interacting partner Dis1 which leads to disruption of MT structure (Fig. 4). Although we have not examined the localisation of any other Ndc80 interactors (e.g. Alp7-Alp14), our results strongly suggest that Ndc80 overproduction leads to spatial segregation of its interacting partners, thereby disrupting the functions of these proteins. To compensate for the loss of function of these proteins, the expression level of these genes may be altered (e.g. up-regulated). Intriguingly, several of the Ndc80 internal loop interactors, such as the Ska complex, TACC-TOG complex and kinesin-8, are also up-regulated in several cancers [3,4,7–9]. Despite this, whether these genes/proteins are coordinately up-regulated with Ndc80 in cancer cells remains to be examined. Further investigations into gene expression profiles of Ndc80 and its interactors will provide insight into the mechanism of Ndc80 overexpression and aneuploidy.

### Does drug usage in combination give a more effective cancer treatment?

Identification of small molecules targeting Ndc80/Hec1 and TACC proteins have given promising results for translation into clinical application [92,94,96,97,99,102]. Oral administrations of these compounds have successfully been shown to reduce tumour growth in vivo, potentially through induction of apoptotic cell death [92,99]. Since recent studies have identified the functional relationships between Ndc80/Hec1 and TACC proteins (see above), we envision that administration of these drugs in combination may have a better effect in cancer treatment. Future studies using cancer cell lines or mouse models will be able to verify this proposition.

## Conclusions and outlook

Genome instability is one of the major aetiologies of tumourigenesis, and is often related to defects in chromosome segregation during mitosis. Due to their roles in chromosome segregation, overexpression of mitotic genes has long been implicated in cancer formation. Although much work has focused on the defining roles of these mitotic genes in chromosome segregation, how the overexpression of these genes leads to tumourigenesis remains largely elusive. Identification of the Ndc80 internal loop as a protein-protein interaction motif has shed light on our understanding of Ndc80 overexpression and cancer formation. We propose that the expression levels of Ndc80-interacting partners may be altered to compensate for loss of functions of these proteins. Further, we propose that our in vivo overproduction system could be exploited as an efficient tool to identify the binding partners of a protein. This tool should provide an alternative way to study genetic and functional relationships between different mitotic genes/proteins. We hope that further investigations into the questions raised in this hypothesis will provide further insights into relationships between gene overexpression and tumourigenesis, leading to improved strategies for cancer treatment.

## Abbreviations

ch-TOG: colonic and hepatic tumour overexpressed gene
CIN: chromosome instability
MAPs: microtubule associated proteins
MTs: microtubules
SAC: spindle assembly checkpoint
SPB: spindle pole body
TACC: transforming acidic coiled-coil
